# Learning Interactions in Reaction Diffusion Equations by Neural Networks

**DOI:** 10.3390/e25030489

**Published:** 2023-03-11

**Authors:** Sichen Chen, Nicolas J-B. Brunel, Xin Yang, Xinping Cui

**Affiliations:** 1 Department of Statistics, University of California, Riverside, CA 92521, USA; 2 ENSIIE & Laboratoire de Mathématiques et Modélisation d’Evry, Université Paris Saclay, 91025 Evry, France; 3 Quantmetry, 75008 Paris, France; 4 Department of Mathematics, University of California, Riverside, CA 92521, USA

**Keywords:** neural networks, deep learning, non-linear reaction–diffusion equations, model discovery, sparse regression, multiple testing

## Abstract

Partial differential equations are common models in biology for predicting and explaining complex behaviors. Nevertheless, deriving the equations and estimating the corresponding parameters remains challenging from data. In particular, the fine description of the interactions between species requires care for taking into account various regimes such as saturation effects. We apply a method based on neural networks to discover the underlying PDE systems, which involve fractional terms and may also contain integration terms based on observed data. Our proposed framework, called Frac-PDE-Net, adapts the PDE-Net 2.0 by adding layers that are designed to learn fractional and integration terms. The key technical challenge of this task is the identifiability issue. More precisely, one needs to identify the main terms and combine similar terms among a huge number of candidates in fractional form generated by the neural network scheme due to the division operation. In order to overcome this barrier, we set up certain assumptions according to realistic biological behavior. Additionally, we use an $L^{2}$-norm based term selection criterion and the sparse regression to obtain a parsimonious model. It turns out that the method of Frac-PDE-Net is capable of recovering the main terms with accurate coefficients, allowing for effective long term prediction. We demonstrate the interest of the method on a biological PDE model proposed to study the pollen tube growth problem.

## 1. Introduction

Two-component reaction–diffusion systems often model the interaction of two chemicals, leading to the formation of non-uniform spatial patterns of chemical concentration or morphogenesis under certain conditions due to chemical reactions and spreading. Since Turing’s groundbreaking work [1], reaction–diffusion systems have been extensively used in developmental biology modeling. For example, let u=u(x,y,t) and v=v(x,y,t) represent the concentration of two chemical species, which may either enhance or suppress each other depending on the context. The system of *u* and *v* can be modeled as follows:

(1) $$\left\{\begin{array}{ccc} \partial_{t}u & = & d_{0}\Delta u+N_{1}\left(u,v\right),\\ \partial_{t}v & = & d_{1}\Delta v+N_{2}\left(u,v\right), \end{array}\right.$$

where $\Delta =\partial_{x}^{2}+\partial_{y}^{2}$ denotes the Laplacian operator, $N_{1}$ and $N_{2}$ are interactions between *u* and *v*. The functions $N_{1}$ and $N_{2}$ are sums of various reaction terms that can be derived from physical or chemical principles such as mass-action laws, Michaelis–Menten kinetics, or products that represent some competition, cooperation effects. We refer the readers to ([2], Section 2.2) for more discussions. Hence, $N_{1}$ and $N_{2}$ are sums of meaningful functions that represent specific mechanisms: if we are able to identify these terms and discover the explicit formulas for $N_{1}$ and $N_{2}$, then we can learn more about the nature of the interactions and predict future behaviors well. This situation arises commonly in biological applications such as chemotaxis, pattern formation in developmental biology, and also the cell polarity phenomenon [3,4].

**Figure 1:**
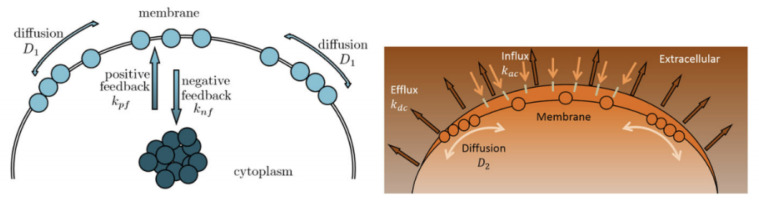
ROP1 and $Ca^{2+}$ polarization dynamics. **Left**: ROP1 dynamics; **Right**: $Ca^{2+}$ dynamics.

Cell polarity plays a vital role in cell growth and function for many cell types, affecting cell migration, proliferation, and differentiation. A classic example of polar growth is pollen tube growth, which is controlled by the Rho GTPase (ROP1) molecular switch. Recent studies have revealed that the localization of active ROP1 is regulated by both positive and negative feedback loops, and calcium ions play a role in ROP1’s negative feedback mechanism. Initially, ROP1 is inside the membrane. During positive feedback (rate $k_{pf}$), some of the ROP1 enters the membrane. At the same time, negative feedback (rate $k_{nf}$) causes some of it to return inside the membrane while the rest diffuse on the membrane (rate $D_{r}$). Calcium ions follow a similar process with positive rate $k_{ac}$, negative rate $k_{dc}$, and diffusion rate $D_{c}$. In [5,6], the following 2D reaction–diffusion system (2) is introduced:

(2) $$\left\{\begin{array}{c} R_{t}=k_{pf}R^{\alpha }\left(R_{tot}-\int_{-L}^{L}R\left(x,t\right)dx\right)-k_{nf}\;g\left(C\right)\;R+D_{r}R_{xx},\\ C_{t}=k_{ac}R-k_{dc}C+D_{c}C_{xx},\\ R_{x}\left(-L,t\right)=R_{x}\left(L,t\right)=0,\;C_{x}\left(-L,t\right)=C_{x}\left(L,t\right)=0,\\ R\left(x,0\right)=R_{0}\left(x\right),\;C\left(x,0\right)=C_{0}\left(x\right). \end{array}\right.$$

with suitable initial and boundary conditions being proposed to quantitatively describe such spatial and temporal connection between ROP1 and calcium ions, leading to rapid oscillations in their distributions on the cell membrane. Here, R=R(x,t), C=C(x,t), and $R_{t}$, $C_{t}$, $R_{x}$, $R_{xx}$, $C_{x}$ and $C_{xx}$ are abbreviated notations for partial derivatives with respect to the time *t* or to the spatial variable *x*. Moreover, the non-linear function g(C) characterizes how calcium ions play a role in ROP1’s negative feedback loop. Specifically, the active ROP1 causes an increase in $Ca^{2+}$ levels, leading to a reduction in ROP1 activity and a decrease in its levels. Meanwhile, the flow of $Ca^{2+}$ slows down as ROP1 drops. Ref. [6] proposed the equation $g\left(C\right)=\frac{C^{2}}{C^{2}+k_{c}^{2}}$ to describe such spatial–temporal patterns of calcium, where $k_{c}$ is a positive constant. Based on this model, ref. [6] developed a modified gradient matching procedure for parameter estimation, including $k_{nf}$ and $k_{c}$. However, it requires that g(C) in (2) is a known function. In this work, we propose to apply neural network methods to uncover the function g(C) or more broadly, to learn interaction terms $N_{1}$ and $N_{2}$ in general reaction-diffusion PDEs (1), which may contain fractional expressions (Figure 1).

In the past decade, the artificial intelligence community has focused increasingly on neural networks, which have become crucial in many applications, especially PDEs. Deep learning-based approaches to PDEs have made substantial progress and are well-studied, both for forward and inverse problems. For forward problems with appropriate initial and boundary conditions in various domains, several methods have been developed to accurately predict dynamics (e.g., [7,8,9,10,11,12,13,14,15,16,17]). For inverse problems, there are two classes of approaches. The first class of approaches focuses on inferring coefficients from known data (e.g., [7,10,12,15,18,19]). An example of this is the widely known PINN (Physics-informed Neural Networks) method [10], which uses PDEs in the loss function of neural networks to incorporate scientific knowledge. Ref. [7] improved the efficiency of PINNs with the residual-based adaptive refinement (RAR) method and created a library of open-source codes for solving various PDEs, including those with complex geometry. However, this method is only capable of estimating coefficients for fixed known terms in PDEs, and may not work well for discovering hidden PDE models. Although [9] extended the PINN method to find unknown dynamic systems, the nonlinear learner function remains a black-box and no explicit expressions of the discovered terms in the predicted PDE are available, making it difficult to interpret their physical meaning. The second class of approaches not only estimates coefficients, but also discovers hidden terms (e.g., [16,17,20,21,22,23,24,25,26]). An example is the PDE-Net method [16], which combines numerical approximations of convolutional differential operators with symbolic neural networks. PDE-Net can learn differential operators through convolution kernels, a natural method for solving PDEs that has been well-studied in [27]. This approach is capable of recovering terms in PDE models with explicit expressions and relatively accurate coefficients, but often produces many noisy terms that lack interpretation. In order to produce parsimonious models, refs. [25,26] proposed to create a regression model with the response variable $\partial_{t}u$, and a matrix Θ with a collection of spatial and polynomial derivative functions (e.g., $u,\partial_{x}u,u\partial_{x}u$): $\partial_{t}u=\Theta \xi$. The estimation of differential equations by modeling the time variations of the solution is known to produce consistent estimates [28]. In addition, the Ridge regression with hard thresholding can be used to approximate the coefficient vector ξ. This sparse regression-based method generally results in a PDE model with accurately predicted terms and high accuracy coefficients. However, few existing studies have focused on effectively recovering interaction terms in the fractional form (say one polynomial term divided by another polynomial term) in hidden partial differential equations, which is the focus of this paper.

Previous methods for identifying the hidden terms in reaction–diffusion partial differential equation models have mostly focused on polynomial forms. However, as indicated in Equation (2), the model for ROP1 and calcium ion distribution also involves fractional and integral forms, which can pose identifiability issues when combined with polynomial forms. Furthermore, we want to attain a parsimonious model, as the interpretability of the PDE model is important for biologists to comprehend biological behavior and phenomena revealed by the model.

In this paper, we utilize a combination of a modified PDE-Net method (which adds fractional and integration terms to the original PDE-Net approach), an $L^{2}$ norm term selection criterion, and an appropriate sparsity regression. This combination proves to produce meaningful and stable terms with accurate estimation of coefficients. For ease of reference to this combination, we call it Frac-PDE-Net.

The paper is organized as follows. In Section 2, we explain the main idea and the framework of our proposed method Frac-PDE-Net. In Section 3, we apply Frac-PDE-Net to discover some biological PDE models based on simulation data. Then, in Section 4, we make some predictions to test the effectiveness of the models learned in Section 3. Finally, we summarize our findings and present some possible future works in Section 5.

## 2. Methodology

The main idea of the PDE-Net method, as described in [16], is to use a deep convolutional neural network (CNN) to study generic nonlinear evolution partial differential equations (PDEs) as shown below:

(3) $$\partial_{t}u=F\left(z,u,\nabla u,\nabla^{2}u,\cdots \right),\;z\in \Omega ,\;\;t\in \left[0,T\right],$$

where u=u(z,t) is a function (scalar valued or vector valued) of the space variable z and the temporal variable *t*. Its architecture is a feed-forward network that combines the forward Euler method in time with the second-order finite difference method in space through the implementation of special filters in the CNN that imitate differential operators. The network is trained to approximate the solution to the above PDEs and then the network is used to make predictions for the subsequent time steps. The authors of [16] show that this approach is effective for solving a range of PDEs and can achieve satisfactory accuracy and computational efficiency compared to traditional numerical methods. In this paper, we follow a similar framework to PDE-Net, but with modifications on a symbolic network framework ($SymNet_{m}^{k}$) to better align with biological models.

### 2.1. PDE-Net Review

The feed-forward network consists of several Δt-blocks, all of which use the same parameters optimized through minimizing a loss function. For simplicity, we will only show one Δt-block for two-dimensional PDEs, as repeating it generates multiple Δt-blocks, and the concept can easily be extended to higher-dimensional PDEs.

Denote the space variable z in (3) to be z=(x,y) since we are dealing with the two-dimensional case. Let $t_{0}=0$ and $\tilde{u}\left(\cdot ,t_{0}\right)$ be the given initial data. For i≥0, $\tilde{u}\left(\cdot ,t_{i+1}\right)$ denotes the predicted value of *u* at time $t_{i+1}$ calculated from the predicted (true) value of $\tilde{u}$ at time $t_{i}$ using the following procedure:

$$\tilde{u}\left(\cdot ,t_{i+1}\right)=\tilde{u}\left(\cdot ,t_{i}\right)+\left(\Delta t\right)\;\mathrm{SymNet}\left(x,y,D_{00}u,D_{10}u,D_{01}u,D_{20}u,\cdots \right),$$

where SymNet is an approximation operator of *F*. Here, the operators $D_{ij}$ are convolution operators with the underlying filters $q_{ij}$, i.e., $D_{ij}u:=\frac{1}{{\left(\Delta x\right)}^{i}{\left(\Delta y\right)}^{j}}\;q_{ij}⊗u$. These operators approximate differential operators:

$$D_{ij}u\approx \frac{d^{i+j}u}{d^{i}xd^{j}y}.$$

For a general N×N filter $q=\left(q[k_{1},k_{2}]\right)$, where $-\frac{N-1}{2}\leq k_{1},k_{2}\leq \frac{N-1}{2}$,

(4) $$q⊗u\left(x,y\right):=\sum_{k_{1},k_{2}}q\left[k_{1},k_{2}\right]\;u\left(x+k_{1}\Delta x,y+k_{2}\Delta y\right).$$

By Taylor expansion,

$$q⊗u\left(x,y\right)=\sum_{i,j=0}^{N-1}m_{ij}{\left(\Delta x\right)}^{i}{\left(\Delta y\right)}^{j}\frac{\partial^{i+j}u}{\partial^{i}x\partial^{j}y}{|}_{\left(x,y\right)}+O\left({\left|\Delta x\right|}^{N}\right)+O\left({\left|\Delta y\right|}^{N}\right),$$

where

$$m_{ij}:=\frac{1}{i!j!}\sum_{k_{1},k_{2}}k_{1}^{i}k_{2}^{j}q\left[k_{1},k_{2}\right],\;\forall \;0\leq i,j\leq N-1.$$

In particular, if choosing Δx=Δy=δ, then

(5) $$q⊗u\left(x,y\right)=\sum_{i,j=0}^{N-1}m_{ij}\;\delta^{i+j}\frac{\partial^{i+j}u}{\partial^{i}x\partial^{j}y}{|}_{\left(x,y\right)}+O\left(\delta^{N}\right),$$

As a result, the training of *q* can be performed through the training of $M:=(m_{ij})$ since the moment matrix M=M(q). It is important to note that the trainable filters *M* (or *q*) must be carefully constrained to match differential operators.

For example, to approximate $\frac{\partial u}{\partial x}$ by $D_{10}u$, or equivalently by $\frac{1}{\Delta x}\;q_{10}⊗u$ for a 3×3 filter $q_{10}$, we may choose

(6) $$\;M_{1}\left(q_{10}\right)=\left(\begin{array}{ccc} 0 & 0 & *\\ 1 & * & *\\ {}* & * & * \end{array}\right)\;\mathrm{or}\;\;M_{2}\left(q_{10}\right)=\left(\begin{array}{ccc} 0 & 0 & 0\\ 1 & 0 & *\\ 0 & * & * \end{array}\right),$$

where ∗ means no constraint on the corresponding entry. Generally, the fewer instances of ∗ present, the more restrictions are imposed, leading to increased accuracy. In this example of (6), the choice of $M_{1}$ ensures the 1st order accuracy and the choice of $M_{2}$ guarantees the 2^*nd*^ order accuracy. More precisely, if we plug $M_{1}$ into (5) with Δx=Δy=δ, then

$$q_{10}⊗u\left(x,y\right)=\delta \frac{\partial u}{\partial x}+O\left(\delta^{2}\right),$$

which implies $\frac{1}{\Delta x}q_{10}⊗u\left(x,y\right)=\frac{\partial u}{\partial x}+O\left(\Delta x\right)$. Similarly, if we plug $M_{2}$ into (5), then $\frac{1}{\Delta x}q_{10}⊗u\left(x,y\right)=\frac{\partial u}{\partial x}+O\left({\left(\Delta x\right)}^{2}\right)$. In PDE-Net 2.0, all moment matrices are trained as subject to partial constraints so that the accuracy is at least 2nd order.

**Figure 2:**
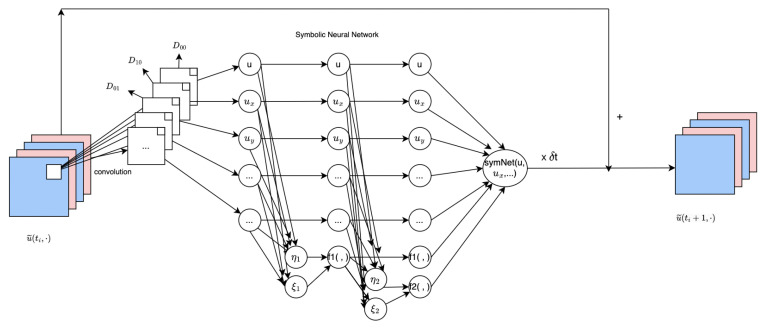
The scheme of one Δt.

The $SymNet_{m}^{k}$ network, modeled after CNNs, is employed to approximate the multivariate nonlinear response function *F*. It takes a *m*-dimensional vector as input and consists of *k* layers. As depicted in Figure 2, the $SymNet_{m}^{2}$ network has two hidden layers, where each $f_{i}$ unit performs a dyadic multiplication and the output is added to the (i+1)th hidden layer.

The loss function for this method has three components and is defined as follows:

(7) $$L=L^{data}+\lambda_{M}L^{moment}+\lambda_{S}L^{SymNet}.$$

Here, $L^{data}$ measures the difference between the true data and the prediction. Consider the data set $\left\{u_{j}\left(\cdot ,t_{i}\right)\in R^{N_{s}\times N_{s}}:1\leq i\leq n,1\leq j\leq N\right\}$, where *n* is the number of Δt blocks, *N* is the total number of samples, and $N_{s}$ is the number of space grids. The index *j* indicates the *j*th solution path with a certain initial condition of the unknown dynamics, and the index *i* represents the solution at time $t_{i}$. Then, we define

$$L^{data}=\frac{1}{nN{\left(\Delta t\right)}^{2}}\sum_{i=1}^{n}\sum_{j=1}^{N}\ell_{ij}.$$

Here, $\ell_{ij}:=\left|\right|u_{j}\left(t_{i},\cdot \right)-{\tilde{u}}_{j}\left(t_{i},\cdot \right){\left|\right|}_{2}^{2}$, where $u_{j}$ represents the real data and ${\tilde{u}}_{j}$ denotes the predicted data. For a given threshold *s*, recall the Huber’s loss function $\ell_{1}^{\left(s\right)}$ defined as

(8) $$\ell_{1}^{\left(s\right)}\left(x\right)=\left\{\begin{array}{ccc} \left|x\right|-\frac{s}{2} & \mathrm{if} & |x|>s,\\ \frac{x^{2}}{2s} & \mathrm{if} & |x|\leq s. \end{array}\right.$$

We then define the following:

$$L^{moment}=\sum_{i,j}\sum_{i_{1},j_{1}}\ell_{1}^{\left(s\right)}\left(M\left(q_{ij}\right)\left[i_{1},j_{1}\right]\right),$$

where $q_{ij}$s are filters and $M(q_{ij})$ is the moment matrix of $q_{ij}$. Using the same Huber loss function as in (8), we define

$$L^{SymNet}=\sum_{i,j}\ell_{1}^{\left(s\right)}\left(w_{ij}\right),$$

where $w_{ij}$s are the parameters in SymNet. The coefficients $\lambda_{M}$ and $\lambda_{S}$ in Equation (7) serve as regularization terms to help control the magnitude of the parameters, preventing them from becoming too large and overfitting to the training data.

### 2.2. mPDE-Net (Modified PDE-Net)

In mPDE-Net, we do not include multiplications between derivatives of *u* and *v*, as these interactions are not commonly present in biological phenomena. Additionally, to handle interactions in fractional or integral forms, such as those in Equation (2), mPDE-Net incorporates integral terms and division operations into $SymNet_{m}^{k}$. However, there was a challenge with identifiability using mPDE-Net. For instance, consider a two-component input vector *u* and *v*. mPDE-Net may produce results such as $\frac{u^{2}}{u+\epsilon }$ or $\frac{uv}{v+\epsilon }$, where ϵ is a small number due to noise. Although both of these terms essentially represent the same term *u*, the mPDE-Net is unable to automatically identify them as such. Keeping all similar terms such as $\frac{u^{2}}{u+\epsilon }$, $\frac{uv}{v+\epsilon }$ and *u* at the same time would result in a complex model and the real fractional term would not be effectively trained.

To address the identifiability issue, restrictions were imposed on the nonlinear interaction term N(u,v) by assuming that N(u,v)=g(u)h(v), where either *g* or *h* is linear and the other one can contain a fractional term with the order of the denominator larger than that of the numerator. For instance, the terms $\frac{u^{2}}{u+\epsilon }$ and $\frac{uv}{v+\epsilon }$ are further decomposed as follows:

$$\frac{u^{2}}{u+\epsilon }=u-\epsilon +\frac{\epsilon^{2}}{u+\epsilon },\;\frac{uv}{v+\epsilon }=u-u\;\frac{\epsilon }{v+\epsilon }.$$

As seen, the main part of the above two terms is *u* while the rest, such as ϵ, $\frac{\epsilon^{2}}{u+\epsilon }$ and $u\frac{\epsilon }{v+\epsilon }$, are considered as perturbations since ϵ is very small. This allows the mPDE-Net to identify and combine the main parts of terms, resulting in a compact model.

**Figure 3:**
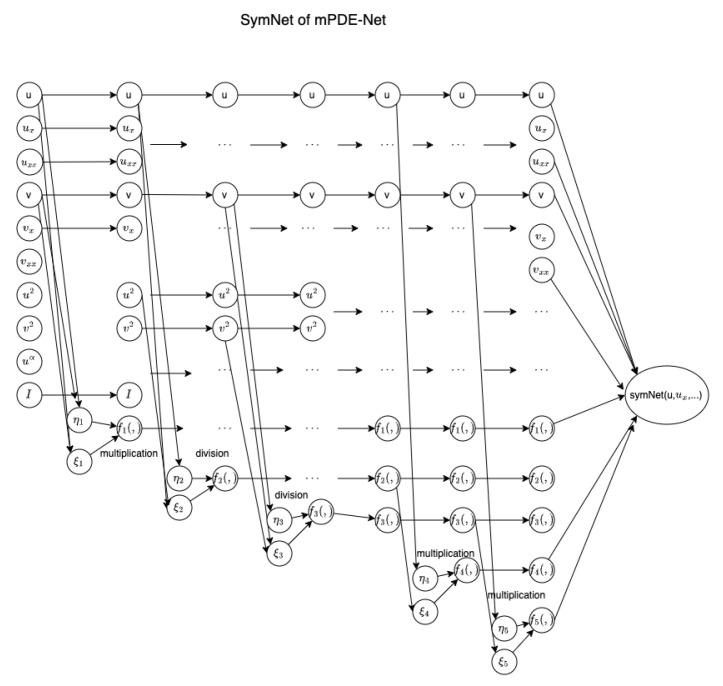
The scheme of mPDE-Net.

Figure 3 presents an example of a system involving the derivatives of *u* and *v* up to the second order. The symbolic neural network in this example has five hidden layers, referred to as $SymNet_{10}^{5}$. The operators $f_{i}$ are multiplication functions, i.e., $f_{i}\left(\eta_{i},\xi_{i}\right)=\eta_{i}\xi_{i}$, for i=1,4,5; and $f_{j}$ are division functions, i.e., $f_{j}\left(\eta_{j},\xi_{j}\right)=\frac{\eta_{j}}{\xi_{j}}$, for j=2,3. Additionally, a term $u^{\alpha }$ is included to incorporate fractional powers, such as the term $R^{\alpha }$ in (2). The algorithm corresponding to this example is outlined in Algorithm 1, where $L_{1}={\left(u,u_{x},u_{xx},v,v_{x},v_{xx},u^{2},v^{2},u^{\alpha },I\right)}^{T},L_{2}={\left(L_{1}^{T},f_{1}\right)}^{T},L_{3}={\left(L_{2}^{T},f_{2}\right)}^{T},L_{4}={\left(L_{3}^{T},f_{3}\right)}^{T},L_{5}={\left(L_{4}^{T},f_{4}\right)}^{T},L_{6}={\left(L_{5}^{T},f_{5}\right)}^{T}.$
**Algorithm** **1** Scheme of mPDE-Net.Input: $u,u_{x},u_{xx},v,v_{x},v_{xx},u^{2},v^{2},u^{\alpha },I$, where I represents ∫u(x,t)dx,${\left(\eta_{1},\xi_{1}\right)}^{T}=W^{\left(1\right)}L_{1}+b^{\left(1\right)}$,    $W^{\left(1\right)}\in R^{2\times 10},\;L_{1}\in R^{10},\;b^{\left(1\right)}\in R^{2}$,${\left(\eta_{2},\xi_{2}\right)}^{T}=W^{\left(2\right)}L_{2}+b^{\left(2\right)}$,    $W^{\left(2\right)}\in R^{2\times 11},\;L_{2}\in R^{11},\;b^{\left(2\right)}\in R^{2}$,${\left(\eta_{3},\xi_{3}\right)}^{T}=W^{\left(3\right)}L_{3}+b^{\left(3\right)}$,    $W^{\left(3\right)}\in R^{2\times 12},\;L_{3}\in R^{12},\;b^{\left(3\right)}\in R^{2}$,${\left(\eta_{4},\xi_{4}\right)}^{T}=W^{\left(4\right)}L_{4}+b^{\left(4\right)}$,    $W^{\left(4\right)}\in R^{2\times 13},\;L_{4}\in R^{13},\;b^{\left(4\right)}\in R^{2}$,${\left(\eta_{5},\xi_{5}\right)}^{T}=W^{\left(5\right)}L_{5}+b^{\left(5\right)}$,    $W^{\left(5\right)}\in R^{2\times 14},\;L_{5}\in R^{14},\;b^{\left(5\right)}\in R^{2}$,Output: $F=W^{\left(6\right)}L_{6}+b^{\left(6\right)}$,    $W^{\left(6\right)}\in R^{1\times 15},\;L_{6}\in R^{15},\;b^{\left(6\right)}\in R^{1}$.

To further demonstrate the mPDE-Net approach, we present a concrete example. To simplify the notation, we introduce the row vector $e_{i}$ with a 1 in the *i*th component and 0 in all other components, i.e.,

$$e_{i}=\left(0,0,\cdots ,0,1,0\cdots ,0\right),$$

where the number “1” is on the *i*th position. Then, we set

$$W^{\left(1\right)}=\left(\begin{array}{c} e_{1}+e_{4}\\ e_{1}+e_{4} \end{array}\right),\;W^{\left(2\right)}=\left(\begin{array}{c} e_{4}\\ 4e_{4}+e_{8} \end{array}\right),\;W^{\left(3\right)}=\left(\begin{array}{c} 0.5e_{1}\\ 0.2e_{1}+e_{7} \end{array}\right),$$

$$W^{\left(4\right)}=\left(\begin{array}{c} 0.2e_{1}\\ e_{12} \end{array}\right),\;W^{\left(5\right)}=\left(\begin{array}{c} 0.2e_{4}\\ e_{13} \end{array}\right),\;W^{\left(6\right)}=0.1e_{1}+0.3e_{3}+6e_{4}+e_{11}+2e_{14}+3e_{15},$$

$$b^{\left(1\right)}=\left(\begin{array}{c} 1\\ 0 \end{array}\right),\;b^{\left(2\right)}=\left(\begin{array}{c} 0.5\\ 0.5 \end{array}\right),\;b^{\left(3\right)}=\left(\begin{array}{c} 1\\ 0 \end{array}\right),\;b^{\left(4\right)}=\left(\begin{array}{c} 0\\ 0 \end{array}\right),\;b^{\left(5\right)}=\left(\begin{array}{c} 0\\ 0 \end{array}\right),\;b^{\left(6\right)}=\left(\begin{array}{c} 0\\ 0 \end{array}\right).$$

According to Algorithm 1 for 1≤i≤5,

$$W^{\left(1\right)}L_{1}+b^{\left(1\right)}=\left(\begin{array}{c} u+v+1\\ u+v \end{array}\right),\;f_{1}=f_{1}\left(\eta_{1},\xi_{1}\right)=\eta_{1}\xi_{1}=\left(u+v+1\right)\left(u+v\right),$$

$$W^{\left(2\right)}L_{2}+b^{\left(2\right)}=\left(\begin{array}{c} v+0.5\\ 4v+v^{2}+0.5 \end{array}\right),\;f_{2}=f_{2}\left(\eta_{2},\xi_{2}\right)=\frac{\eta_{2}}{\xi_{2}}=\frac{v+0.5}{v^{2}+4v+0.5},$$

$$W^{\left(3\right)}L_{3}+b^{\left(3\right)}=\left(\begin{array}{c} 0.5u+1\\ 0.2u+u^{2} \end{array}\right),\;f_{3}=f_{3}\left(\eta_{3},\xi_{3}\right)=\frac{\eta_{3}}{\xi_{3}}=\frac{0.5u+1}{u^{2}+0.2u},$$

$$W^{\left(4\right)}L_{4}+b^{\left(4\right)}=\left(\begin{array}{c} 0.2u\\ f_{2} \end{array}\right),\;f_{4}=f_{4}\left(\eta_{4},\xi_{4}\right)=\eta_{4}\xi_{4}=0.2u\;f_{2}=0.2u\;\frac{v+0.5}{v^{2}+4v+0.5},$$

$$W^{\left(5\right)}L_{5}+b^{\left(5\right)}=\left(\begin{array}{c} 0.2v\\ f_{3} \end{array}\right),\;f_{5}=f_{5}\left(\eta_{5},\xi_{5}\right)=\eta_{5}\xi_{5}=0.2v\;f_{3}=0.2v\;\frac{0.5u+1}{u^{2}+0.2u},$$

Therefore,

$$\begin{array}{cc} SymNet_{10}^{5} & =W^{6}L_{6}+b^{\left(6\right)}=0.1u+0.3u_{xx}+6v+f_{1}+2f_{4}+3f_{5}\\ & =0.3u_{xx}+u^{2}+2uv+v^{2}+1.1u+7v+0.4u\;\frac{v+0.5}{v^{2}+4v+0.5}+0.6v\;\frac{0.5u+1}{u^{2}+0.2u}. \end{array}$$

Let L denote the library for PDE-Net 2.0 and $L_{f}$ denote the library for mPDE-Net. It is clear that L and $L_{f}$ are distinct. Typically, L only seeks to identify multiplication terms and has the form:

$$L=\left\{\lambda \left(U_{xx}+U_{yy}\right)+f_{1}\left(U\right):\;\lambda \in R,\;U=\left(u,v\right),\;f_{1}\in P\right\},$$

where

P:={Polynomials of Uup to a certain degree}.

Conversely, $L_{f}$ is engineered to learn both multiplication terms and fractional terms, subject to certain constraints. In our paper, we make the choice of

$$\begin{array}{cc} L_{f}= & \{\lambda \left(U_{xx}+U_{yy}\right)+f_{1}\left(U\right)+\frac{f_{2}\left(u\right)}{f_{3}\left(u\right)}f_{4}\left(v\right)+f_{5}\left(u\right)\frac{f_{6}\left(v\right)}{f_{7}\left(v\right)}:\\ & \;\lambda \in R,\;U=\left(u,v\right),\;{\left\{f_{i}\right\}}_{i=1}^{7}\subset P,\;\deg f_{2}<\deg f_{3},\;\deg f_{6}<\deg f_{7}\}, \end{array}$$

which is much larger than L. Therefore, our framework of neural networks, built upon $L_{f}$, is more challenging to implement than the original framework, which is based on L.

### 2.3. Optimizing Hyperparameters

In this section, we will explain the process of tuning hyperparameters $\lambda_{M}$ and $\lambda_{S}$ in the loss function (7). Firstly, the range of spatial and temporal variables in the training set are defined as [−L,L] and [0,T], respectively. Then, using the finite difference method, we generate a dataset that acts as the “true data”. Additionally, we consider *M* initial conditions. The time interval is determined by $dt/\tilde{d}t$, where $\tilde{d}t$ is the time step size for computing the “true data” and dt represents the time step size for selecting the “observational data”. Typically, $\tilde{d}t$ is chosen to be much smaller than dt. The solution corresponding to the *m*th initial condition is denoted as $u_{m}\left(\cdot ,\cdot \right)$, where the first “·” refers to the spatial variable and the second “·” represents the temporal variable. If the solution is evaluated at the *k*th time step, it is written as $u_{m}\left(\cdot ,t_{k}\right)$, with “·” representing the spatial variable.

The *M* initial values from *M* initial conditions are divided into three separate groups, resulting in $M=M_{1}+M_{2}+M_{3}$, where $M_{1}$, $M_{2}$, and $M_{3}$ represent the sizes of the training set, validation set, and test set, respectively. The solutions produced by these initial values are designated as follows:

Training set: $u_{1}\left(\cdot ,\cdot \right),\cdots ,u_{M_{1}}\left(\cdot ,\cdot \right)$;

Validation set: $u_{M_{1}+1}\left(\cdot ,\cdot \right),\cdots ,u_{M_{1}+M_{2}}\left(\cdot ,\cdot \right)$;

Testing set: $u_{M_{1}+M_{2}+1}\left(\cdot ,\cdot \right),\cdots ,u_{M_{1}+M_{2}+M_{3}}\left(\cdot ,\cdot \right)$.

We use the training set to train our models, the validation set to find the best parameters, and the testing set to evaluate the performance of the trained models.

Assume we divide the time range [0,T] into *K* blocks, with cutting points denoted as $t_{k}$ for 1≤k≤K. Then, for any 1≤m≤M and for any 1≤k≤K, we define

$$\ell_{km}=\left|\right|u_{m}\left(\cdot ,t_{k}\right)-{\tilde{u}}_{m}\left(\cdot ,t_{k}\right){\left|\right|}_{2}^{2},$$

where ${∥\cdot ∥}_{2}$ denotes the $L^{2}$ norm with respect to the space variable on [−L,L], $u_{m}$ is the “true solution”, and ${\tilde{u}}_{m}$ is the “predicted solution” by a neural network. Based on this, the training loss, validation loss and the testing loss are defined as follows:

- Training loss:
  (9) $$L_{train}:=\frac{1}{M_{1}K{\left(dt\right)}^{2}}\sum_{k=1}^{K}\sum_{m=1}^{M_{1}}\ell_{km}.$$
- Validation loss:
  (10) $$L_{valid}:=\frac{1}{M_{2}K{\left(dt\right)}^{2}}\sum_{k=1}^{K}\sum_{m=M_{1}+1}^{M_{1}+M_{2}}\ell_{km}.$$
- Testing loss:
  (11) $$L_{test}:=\frac{1}{M_{3}K{\left(dt\right)}^{2}}\sum_{k=1}^{K}\sum_{m=M_{1}+M_{2}+1}^{M}\ell_{km}.$$

We choose the hyperparameters $\lambda_{M}$ and $\lambda_{S}$ in the loss function (7) using the validation sets. Let $B_{mk}=u_{m}\left(\cdot ,t_{k}\right)$ and $B_{jk}=u_{j}\left(\cdot ,t_{k}\right)$, where $1\leq m\leq M_{1}$, $M_{1}+1\leq j\leq M_{1}+M_{2}$ and 1≤k≤K. We define the training number by $N_{t}$. We then gradually increase the time points of the training and validation sets. For instance, if K = 15 and $N_{t}=5$, the training and validation sets can be selected as follows. The performance metric is the same as the validation loss in (10).
Training       Validation       Validation Loss$B_{m1},\cdots ,B_{m3}$
$B_{j1},\cdots ,B_{j3}$
$L_{valid}^{\left(1\right)}$$B_{m1},\cdots ,B_{m6}$
$B_{j1},\cdots ,B_{j6}$
$L_{valid}^{\left(2\right)}$$B_{m1},\cdots ,B_{m9}$
$B_{j1},\cdots ,B_{j9}$
$L_{valid}^{\left(3\right)}$$B_{m1},\cdots ,B_{m12}$
$B_{j1},\cdots ,B_{j12}$
$L_{valid}^{\left(4\right)}$$B_{m1},\cdots ,B_{m15}$
${\tilde{B}}_{j1},\cdots ,B_{j15}$
$L_{valid}^{\left(5\right)}$

Furthermore, we tune the hyperparameters using Hyperopt [29], which uses Bayesian optimization to explore the hyperparameter space more efficiently than a brute-force grid search. Specifically, the mPDE-Net is nested in the objective function of Hyperopt, which will optimize the average validation loss $L_{avl}$ of models.

$$L_{avl}=\frac{1}{5}\sum_{i=1}^{5}L_{valid}^{\left(i\right)}.$$

The selection procedure is described in Algorithm 2.
**Algorithm** **2** Optimizing Hyperparameters using Hyperopt1:Initialize the search spaces for $\lambda_{M}$ and $\lambda_{S}$;2:Define the objective function (to be optimized) as the average testing loss obtained from mPDE-Net, implemented using PyTorch;3:Set the optimization algorithm, specify the number of trials, and initialize the results list.4:**for** i=1 to number of trials **do**5:      Sample a set of hyperparameters from the search spaces, evaluate the objective function with the sampled hyperparameters, and set a list called the Validation loss.6:      **for** r=1 to $\frac{K}{N_{t}}$ **do**7:        Train model of mPDE-Net on $B_{m1},\cdots ,B_{mr}$, test it on $B_{j1},\cdots ,B_{jr}$ to get a validation loss, and then append the validation loss to the Validation loss.8:      **end for**9:      Get an average validation loss from the Validation loss, append the hyperparameters and the average validation loss to the results list, and then update the search space based on the results so far.10:**end for**11:**return** the hyperparameters with the minimum objective function value.

### 2.4. Frac-PDE-Net

We have noted that mPDE-Net accurately fits data and recovers terms, but it may not always simplify the learned PDE, making it challenging to interpret. To address this, we implement sparsity-encouraging methods such as the Lasso approach. However, even with Lasso and hyperparameters chosen from the validation sets, the predicted equation still had redundant terms. This is likely due to correlated data and linear dependencies in the data, which prevent Lasso from fully shrinking the extra coefficients to zero. To overcome this, we employ two approaches. The first, called the $L^{2}$ norm-based term selection criterion, weakens or eliminates linear dependencies in the data. The second, called sequential threshold ridge regression (STRidge), creates concise models through strong thresholding. We will discuss these approaches in more detail below.

**$L^{2}$ norm based term selection criterion.** Consider the underlying PDE in the form of
  (12) $$\partial_{t}u=\Theta \left(u\right)\xi ,$$
  where
  $$\Theta \left(u\right)=\left(\Theta_{1}\left(u\right),\Theta_{2}\left(u\right),\cdots ,\Theta_{p}\left(u\right)\right),\;\xi ={\left(\xi_{1},\xi_{2},\cdots ,\xi_{p}\right)}^{T}.$$
  To address the issue of excessive terms in the learned PDE, we apply the $L^{2}$ norm based term selection criterion. This involves normalizing the columns of Θ(u) to obtain $\Phi_{k}\left(u\right)$
  $$\Theta \left(u\right)\xi =\sum_{k=1}^{p}\Theta_{k}\left(u\right)\xi_{k}=\sum_{k=1}^{p}\Phi_{k}\left(u\right)\eta_{k},$$
  where
  $$\Phi_{k}\left(u\right)=\frac{\Theta_{k}\left(u\right)}{∥\Theta_{k}{\left(u\right)∥}_{2}},\;\eta_{k}=\xi_{k}{∥\Theta_{k}\left(u\right)∥}_{2},\;\forall \;1\leq k\leq p,$$
  and adjusting the coefficients ξ to $\tilde{\xi }$,
  $${\tilde{\xi }}_{k}=\left\{\begin{array}{cc} 0, & \mathrm{if}\;|\eta_{k}|<\delta \max_{j}\left|\eta_{j}\right|,\\ \xi_{k}, & \mathrm{otherwise}, \end{array}\right.\;\forall \;1\leq k\leq p.$$
  By removing the terms in Θ(u) whose adjusted coefficients $\eta_{k}$ are significantly smaller than the largest one, we shorten the vector $\tilde{\xi }$ to $\xi^{\left(s\right)}$. The corresponding columns in Θ(u) form a new matrix $\Theta^{\left(s\right)}\left(u\right)$ with reduced linear dependency between its columns. This results in a simplified approximation of the PDE:
  (13) $$\partial_{t}u\approx \Theta^{\left(s\right)}\left(u\right)\;\xi^{\left(s\right)}.$$
**Sparse regression: STRidge.** After using the $L^{2}$ norm-based term selection criterion to select terms, as discussed previously, we move on to consider sparse regression to further improve the compactness of the representation for the hidden PDE model (13). Here, a tolerance threshold “tol” is introduced to select coefficients for sparse results. Coefficients smaller than “tol” will be discarded, and the remaining ones will be utilized until the number of terms stabilizes. The sparsity regression process is outlined in Algorithm 3. For further information, see [25].

To summarize, the mPDE-Net approach allows us to achieve relatively accurate predictions for the function and its derivatives. We then employ an $L^{2}$ norm-based term selection criterion and sparse regression to obtain a concise model, which we refer to as Frac-PDE-Net. Algorithm 4 summarizes this procedure.
**Algorithm 3**: STRidge($\Theta^{\left(s\right)}$, $U_{t}$, λ, tol, iters)1:${\hat{\xi }}^{\left(s\right)}=argmin_{\xi^{\left(s\right)}}\left|\right|\Theta^{\left(s\right)}\xi^{\left(s\right)}-U_{t}{\left|\right|}_{2}^{2}+\lambda ||\xi^{\left(s\right)}{\left|\right|}_{2}^{2}$                      ▹ ridge regression2:bigcoeffs=$\left\{j:|{\hat{\xi }}_{j}^{\left(s\right)}|\geq tol\right\}$                              ▹ select large coefficients3:${\hat{\xi }}^{\left(s\right)}\left[∼\mathrm{bigcoeffs}\right]=0$                                 ▹ apply hard threshold4:${\hat{\xi }}^{\left(s\right)}\left[\mathrm{bigcoeffs}\right]=STRidge\left(\Theta^{\left(s\right)}\left[:,\mathrm{bigcoeffs}\right],U_{t},\lambda ,\mathrm{tol},\mathrm{iters}-1\right)$    ▹ recursive call with fewer coefficients5:return ${\hat{\xi }}^{\left(s\right)}$

**Algorithm 4**: $L^{2}$ Norm selection criterion+ STRidge($\hat{\Theta }$, ${\hat{u}}_{t}$, λ, tol, iters)
1:$\hat{\Theta }\hat{\xi }=\sum_{k=1}^{p}{\hat{\Theta }}_{k}{\hat{\xi }}_{k}=\sum_{k=1}^{p}\frac{\Theta_{k}\left(\hat{u}\right)}{∥\Theta_{k}\left(\hat{u}\right){∥}_{2}}\left({\hat{\xi }}_{k}{∥\Theta_{k}\left(\hat{u}\right)∥}_{2}\right)=\sum_{k=1}^{p}\Phi_{k}\left(\hat{u}\right)\eta_{k}$           ▹ Adjusted coefficients2:bigcoeffs=$\left\{k:|\eta_{k}|\geq \delta \max_{j}|\eta_{j}|\right\}$                            ▹ Select large coefficients3:

$\hat{\xi }\left[∼\mathrm{bigcoeffs}\right]=0$

4:$\Theta^{\left(s\right)}=\hat{\Theta }\left[:,\mathrm{bigcoeffs}\right]$ and $\xi^{\left(s\right)}=\hat{\xi }\left[\mathrm{bigcoeffs}\right]$5:${\hat{\xi }}^{\left(s\right)}=argmin_{\xi^{\left(s\right)}}\left|\right|\Theta^{\left(s\right)}\xi^{\left(s\right)}-{\hat{u}}_{t}{\left|\right|}_{2}^{2}+\lambda ||\xi^{\left(s\right)}{\left|\right|}_{2}^{2}$▹ ridge regression6:bigcoeffs=$\left\{j:|{\hat{\xi }}_{j}^{\left(s\right)}|\geq tol\right\}$                               ▹ select large coefficients7:${\hat{\xi }}^{\left(s\right)}\left[∼\mathrm{bigcoeffs}\right]=0$                                      ▹ apply hard threshold8:${\hat{\xi }}^{\left(s\right)}\left[\mathrm{bigcoeffs}\right]=STRidge\left(\Theta^{\left(s\right)}\left[:,\mathrm{bigcoeffs}\right],{\hat{u}}_{t},\lambda ,\mathrm{tol},\mathrm{iters}-1\right)$  ▹ recursive call with fewer non-zero coefficients9:return ${\hat{\xi }}^{\left(s\right)}$


### 2.5. Kolmogorov-Smirnov Test

After applying the Frac-PDE-Net procedure, a simplified, interpretable model has been created. Our next goal is to determine if this model can be further compressed. We designate Model 1 as the system learned by Frac-PDE-Net, and Model 2 as the system obtained by removing the interaction term with the smallest $L^{2}$ norm from Model 1. To determine if Model 1 and Model 2 come from the same distribution, we use the Kolmogorov–Smirnov test (K-S test).

Since our examples involve systems of two PDEs, a two-dimensional K-S test is appropriate. The time range is [0,T] with time step size dt, giving $n:=\frac{T}{dt}$ time grids denoted as ${\left\{t_{i}\right\}}_{i=1}^{n}$, where $t_{i}=i\left(dt\right)$, and 1≤i≤n. At a fixed time $t_{i}$, we aim to test the proximity of two samples $Y_{t_{i}}$ and ${\tilde{Y}}_{t_{i}}$, which are associated with Model 1 and Model 2, respectively, at time $t_{i}$. For each $t_{i}$, we specify:

**Hypothesis** **1** (Null)**.** *The two sets ${\left\{Y_{t_{i}}\right\}}_{i=1}^{n}$ and ${\left\{{\tilde{Y}}_{t_{i}}\right\}}_{i=1}^{n}$ come from a common distribution.*

**Hypothesis** **2** (Alternative)**.** *The two sets ${\left\{Y_{t_{i}}\right\}}_{i=1}^{n}$ and ${\left\{{\tilde{Y}}_{t_{i}}\right\}}_{i=1}^{n}$ do not come from a common distribution.*

Let $H_{t_{i},0}$ and ${\hat{p}}_{t_{i}}$ denote null hypotheses and the corresponding p-values, respectively, for 1≤i≤n. In this paper, we employed Bonerroni [30], Holm [31] and Benjamini–Hochberg (B-H) [32] methods for multiple testing adjustment. Note that the Bonferroni method is the most conservative one among these three methods. Under the complete null hypothesis of a common distribution across all time points, no more than 5% of the total time points can be rejected.

## 3. Numerical Studies: Convection-Diffusion Equations with the Neumman Boundary Condition

In this section, we showcase numerical examples to demonstrate the efficacy of Frac-PDE-Net, our proposed method. The training, validation, and testing data are generated based on the underlying governing equation. Our aim is to use Frac-PDE-Net on these data to obtain a concise and interpretable model for the PDE. The governing PDEs under consideration in this paper are of the following form:

(14) $$\left\{\begin{array}{c} \partial_{t}u=F_{1}\left(u,v\right),\\ \partial_{t}v=F_{2}\left(u,v\right), \end{array}\right.$$

where

(15) $$F_{1}\left(u,v\right)=d_{1}\Delta u+P_{1}\left(u,v\right)+R_{1}\left(u,v\right),\;F_{2}\left(u,v\right)=d_{2}\Delta v+P_{2}\left(u,v\right)+R_{2}\left(u,v\right).$$

Here, $d_{1}$ and $d_{2}$ are positive diffusion coefficients, $R_{1}$ and $R_{2}$ represent fractional functions of (u,v), and $P_{1}$ and $P_{2}$ denote combinations of power functions and integration operators of (u,v) through addition and multiplication. For example, $R_{1}\left(u,v\right)$ can be $\frac{u-2}{v^{2}-v+3}$, and $P_{1}\left(u,v\right)$ can be $1+u^{1.5}-v^{2}+u^{1.5}\int u\;dx$.

### 3.1. Example 1: A 2-Dimensional Model

Our first example is taken from (Equation (2.8) in Section 2.2 in [2]). In this example, we consider (14) under the Neumann boundary condition on a 2-dimensional domain $D_{1}:=\left[-5,5\right]\times \left[-5,5\right]$ with $d_{1}=0.3$, $d_{2}=0.4$, $P_{1}\left(u,v\right)=1-u$, $P_{2}\left(u,v\right)=0.4-0.2v$, and

$$R_{1}\left(u,v\right)=R_{2}\left(u,v\right)=-2\;\frac{uv}{1+u+4u^{2}}=-\frac{1}{2}\;\frac{uv}{u^{2}+0.25u+0.25}.$$

Thus, Equation (14) is reduced to

(16) $$\left\{\begin{array}{c} \partial_{t}u=F_{1}\left(u,v\right),\\ \partial_{t}v=F_{2}\left(u,v\right),\\ \partial_{x}u\left(-5,y,t\right)=\partial_{x}u\left(5,y,t\right)=\partial_{y}u\left(x,-5,t\right)=\partial_{y}u\left(x,5,t\right)=0, \end{array}\right.$$

with (x,y,t)∈[−5,5]×[−5,5]×[0,0.15] and

(17) $$\left\{\begin{array}{ccc} F_{1}\left(u,v\right) & = & 0.3\left(\partial_{x}^{2}u+\partial_{y}^{2}u\right)+1-u-\frac{1}{2}\;\frac{uv}{u^{2}+0.25u+0.25},\;\\ F_{2}\left(u,v\right) & = & 0.4\left(\partial_{x}^{2}v+\partial_{y}^{2}v\right)+0.4-0.2v-\frac{1}{2}\;\frac{uv}{u^{2}+0.25u+0.25} \end{array}\right.$$

The observations are generated with Equations (16) and (17), and then split into training data, validation data and testing data. The PDE is solved by applying a finite difference scheme to a 64×64 spatial mesh grid with the central difference scheme for $\Delta :=\partial_{x}^{2}+\partial_{y}^{2}$, and with a temporal discretization of second-order Runge–Kutta (see [16]), using a time step size of $\frac{1}{1600}$.

In addition, the observations are obtained from various initial values: this implies an extra variability in the datasets, that is necessary if we want to be able to generalize well to any initial conditions. We assume that we have $N_{Init}=12$ different solutions, coming from different initial values $w_{0}$. The functions are random, defined through random parameters $a_{i,j}$, $b_{i,j}$, $c_{i,j}$, $d_{i,j}$, $a_{k,l}$, $b_{k,l}$, $c_{k,l}$ and $d_{k,l}$, which follow from the standard normal distribution N(0,1), $c_{1}$ and $c_{2}$, which follow from uniform distributions: $c_{1}∼U\left(-0.5,0.5\right)$ and $c_{2}∼U\left(0.5,1.5\right)$. Then, we generate the 12 initial values $\left(u_{0},v_{0}\right)$ by setting

(18) $$u_{0}\left(x,y\right)=\frac{w_{0}\left(x,y\right)}{\max|w_{0}|}+c_{1},\;v_{0}\left(x,y\right)=\frac{{\tilde{w}}_{0}\left(x,y\right)}{\max|{\tilde{w}}_{0}|}+c_{2},$$

where

$$\begin{array}{cc} w_{0}\left(x,y\right)=\sum_{|i|,|j|\leq 13} & \{a_{i,j}\cos\left(2ix\right)\cos\left(2jy\right)+b_{i,j}\sin\left[\left(2i+1\right)x\right]\sin\left[\left(2j+1\right)y\right]\\ & \;+c_{i,j}\sin\left[\left(2i+1\right)x\right]\cos\left(2jy\right)+d_{i,j}\cos\left(2ix\right)\sin\left[\left(2j+1\right)y\right]\}, \end{array}$$

$$\begin{array}{cc} {\tilde{w}}_{0}\left(x,y\right)=\sum_{|k|,|l|\leq 13} & \{a_{k,l}\cos\left(2kx\right)\cos\left(2ly\right)+b_{k,l}\sin\left[\left(2k+1\right)x\right]\sin\left[\left(2l+1\right)y\right]\\ & \;+c_{k,l}\sin\left[\left(2k+1\right)x\right]\cos\left(2ly\right)+d_{k,l}\cos\left(2kx\right)\sin\left[\left(2l+1\right)y\right]\}. \end{array}$$

For any given initial data $\left(u_{0},v_{0}\right)$, we denote the corresponding solution to be $\left(u^{*},v^{*}\right)$. When noise is allowed, we assume the perturbed data to be

$$u\left(x,y,t\right)=u^{*}\left(x,y,t\right)+n_{l}Q_{1},\;v\left(x,y,t\right)=v^{*}\left(x,y,t\right)+n_{l}Q_{2},$$

where $n_{l}$ is the level of Gaussian noise added, and $Q_{1}$ and $Q_{2}$ are random variables, which follow from the normal distribution: $Q_{i}∼N\left(0,\sigma_{i}^{2}\right)$ for i=1,2, where $\sigma_{1}$ (or $\sigma_{2}$ resp.) is the standard deviation of the true data $u^{*}$ (or $v^{*}$ resp.).

Since the time is from 0 to 0.15, there are 15 time blocks and we denote $N_{Time}=15$. For spatial variables, we have $N_{Space}=64$, where $N_{Space}$ represents the number of space grids. Therefore, the dataset is

$$\left\{\left(u_{t,k},v_{t,k}\right):1\leq t\leq N_{Time},\;1\leq k\leq N_{Init}\right\},$$

where both $u_{t,k}$ and $v_{t,k}$ are matrices in $R^{N_{Space}\times N_{Space}}$. The following Table 1 and Table 2 show a summary of parameters for Frac-PDE-Net.

Our goal is to discover the terms $F_{1}\left(u,v\right)$ and $F_{2}\left(u,v\right)$ on the right hand side of (16) and the true expressions are given by (17). For convenience of notation, we denote ${\hat{F}}_{1}$ and ${\hat{F}}_{2}$ to be our predicted operators for $F_{1}$ and $F_{2}$. Based on some existing models (see, e.g., Section 2.2 in [2]), we adopt some assumptions before discovering ${\hat{F}}_{1}$ and ${\hat{F}}_{2}$. More precisely, we assume that

(19) $${\hat{F}}_{1}\left(u,v\right)={\hat{d}}_{1}\Delta u+{\hat{P}}_{1}\left(u,v\right)+{\hat{R}}_{1}\left(u,v\right),\;{\hat{F}}_{2}\left(u,v\right)={\hat{d}}_{2}\Delta v+{\hat{P}}_{2}\left(u,v\right)+{\hat{R}}_{2}\left(u,v\right),$$

where ${\hat{d}}_{1}$ and ${\hat{d}}_{2}$ are positive constants, ${\hat{P}}_{1}$ and ${\hat{P}}_{2}$ are polynomials of (u,v) up to order 2, and both the fractional terms ${\hat{R}}_{1}$ and ${\hat{R}}_{2}$ are in the form l(u)r(v) or r(u)l(v), where *l* means a linear function and *r* denotes a fractional function in which the numerator is linear and the denominator is quadratic.

Based on these assumptions, we consider the following library $\left\{u,\;u_{xx},\;u_{yy},\;v,\;v_{xx},\;v_{yy}\right\}$ for training our model.

The filters *q* (as defined in (4)) are selected to be of size 5×5. The total number of parameters in $W^{\left(i\right)}$ (as defined in Algorithm 1) for approximating $F_{1}$ and $F_{2}$ is 56, and the number of trainable parameters in the moment matrices *M* (as defined in (6)) is 52. To optimize the parameters, we use the BFGS algorithm instead of the Adam or SGD optimizers since the BFGS algorithm is faster and also stable.

In the following, we outline the notation used and summarize the key steps of our framework.

1. ${\hat{F}}_{i}^{mPDE-Net}$ denotes the result of applying the modified PDE-Net on our model.
2. Next, we utilize the $L^{2}$ norm-based selection criterion and sparse regression on ${\hat{F}}_{i}^{mPDE-Net}$ to obtain a more concise and interpretable model, referred to as ${\hat{F}}_{i}^{smPDE-Net}$. The “s” in ${\hat{F}}_{i}^{smPDE-Net}$ represents the application of sparse regression.
3. Subsequently, we fix the terms in ${\hat{F}}_{i}^{smPDE-Net}$ and retrain its coefficients to produce a final model named ${\hat{F}}_{i}^{rsmPDE-Net}$. This is the end result of our Frac-PDE-Net scheme. The “r” in ${\hat{F}}_{i}^{rsmPDE-Net}$ signifies the process of retraining the coefficients.
4. Finally, to verify that no further terms can be eliminated after Frac-PDE-Net, we compare two models: Model 1, generated by Frac-PDE-Net; and Model 2, which is identical to Model 1 but removes the term with the smallest $L^{2}$ norm from ${\hat{F}}_{1}$ and ${\hat{F}}_{2}$. The coefficients in Model 2 are retrained, and the resulting model is referred to as ${\hat{F}}_{i}^{PHrsmPDE-Net}$. “PH” in ${\hat{F}}_{i}^{PHrsmPDE-Net}$ represents the Post-hoc selection in Model 2. The comparison between Model 1 and Model 2 will be conducted using the Kolmogorov–Smirnov test as outlined in Section 2.5.

For this case, we added 5% noise to the generated data to form the observational data. The results are displayed in Table 3. Table 3 shows that ${\hat{F}}_{i}^{mPDE-Net}$ (modified PDE-Net framework) accurately identifies the terms in Example 1 and estimates their corresponding coefficients. However, it also produces unnecessary terms with low weights after training. By applying the $L^{2}$ norm-based selection and sparse regression ($L^{2}$+SP), we successfully remove these extra terms in ${\hat{F}}_{i}^{rsmPDE-Net}$. After the terms in ${\hat{F}}_{1}$ and ${\hat{F}}_{2}$ are identified, we retrain the model with these fixed terms to obtain the final coefficients in ${\hat{F}}_{i}^{rsmPDE-Net}$.

To test whether Model 1 (${\hat{F}}_{i}^{rsmPDE-Net}$) and Model 2 (${\hat{F}}_{i}^{PHrsmPDE-Net}$) are similar or not, we compare their predictions by using the finite difference scheme. Consider the time range to be [0,0.5] with time step size dt=0.01. Hence, there are 50 time grids, which are denoted to be ${\left\{t_{i}\right\}}_{i=1}^{50}$, where $t_{i}=0.01i$, 1≤i≤50. Fix a time $t_{i}$, we introduce the residuals $E_{t_{i}}:=Y_{t_{i}}-Y_{t_{i}}^{*}$ and ${\tilde{E}}_{t_{i}}:={\tilde{Y}}_{t_{i}}-Y_{t_{i}}^{*}$, where $Y_{t_{i}}^{*}$ represents the true solution, and $Y_{t_{i}}$ and ${\tilde{Y}}_{t_{i}}$ denote the predicted solutions based on Model 1 and Model 2, respectively, at time $t_{i}$. We will test if the residuals ${\left\{E_{t_{i}}\right\}}_{i=1}^{50}$ and ${\left\{{\tilde{E}}_{t_{i}}\right\}}_{i=1}^{50}$ have similar distributions. The null hypothesis is $H_{0}^{\left(i\right)}:E_{t_{i}}∼{\tilde{E}}_{t_{i}}$ and the alternative hypothesis is $H_{A}^{\left(i\right)}:E_{t_{i}}≁{\tilde{E}}_{t_{i}}$. Applying Bonferroni method, Holm method and the B-H’s procedure for multiple testing adjustment, discussed in Section 2.5, the test results are presented in the following Table 4.

The results in Table 4 show that Model 1 (Frac-PDE-Net) is significantly different from Model 2, meaning all terms in Model 1 should be kept. Hence, the final discovered terms for ${\hat{F}}_{1}$ and ${\hat{F}}_{2}$ are represented by Model 1 (Frac-PDE-Net) in Table 4.

**Figure 4:**
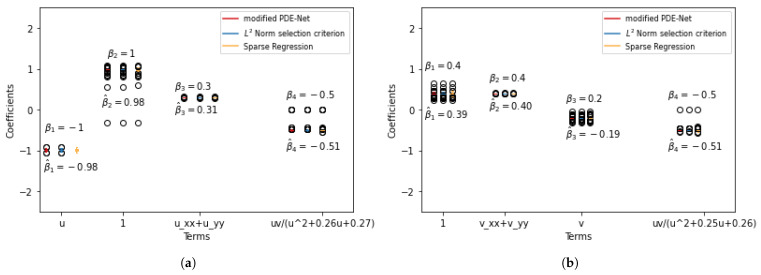
Simulation results for true positive discovering with 5% noise. (**a**) ${\hat{F}}_{1}$. (**b**) ${\hat{F}}_{2}$.

**Figure 5:**
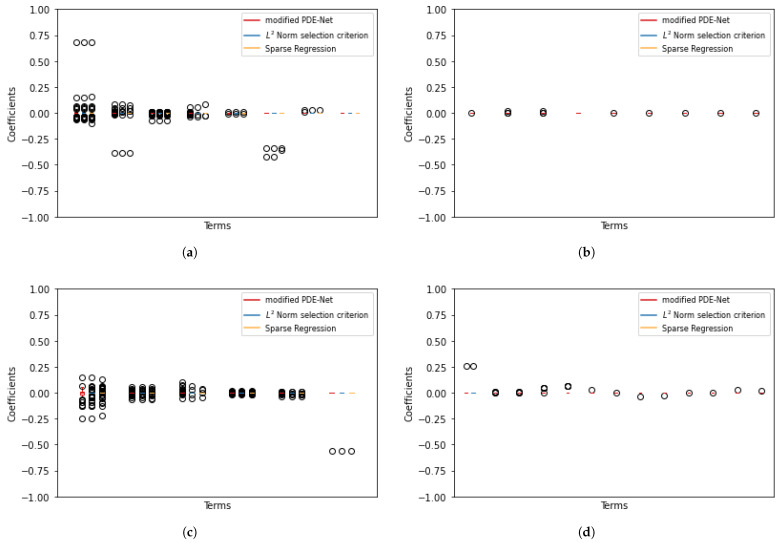
Simulation results for false positive discovering with 5% noise. (**a**) ${\hat{F}}_{1}$. (**b**) ${\hat{F}}_{1}$. (**c**) ${\hat{F}}_{2}$. (**d**) ${\hat{F}}_{2}$.

To assess the stability of the results shown above, we repeated the experiments 100 times and the results are presented in Figure 4 and Figure 5. The process of merging similar terms is outline in Appendix A.1. The plots show that there are some instances where the three methods fail to eliminate certain redundant terms. However, these instances are rare, as the median of these terms is 0, indicating that they appear infrequently.

### 3.2. Example 2: A 1-Dimensional Model

Our second example is taken from [6]. In this example, we consider (14) under the Neumann boundary condition on a one-dimensional domain $D_{1}:=\left[-\frac{5\pi }{2},\frac{5\pi }{2}\right]$ with $d_{1}=0.1,d_{2}=10$,

$$P_{1}\left(u,v\right)=3.6u^{1.5}-3.6u-0.229u^{1.5}\int_{-2.5\pi }^{2.5\pi }u\;dx,\;P_{2}\left(u,v\right)=u-0.4v,$$

$$R_{1}\left(u,v\right)=0.081\;\frac{u}{v^{2}+0.0215},\;R_{2}\left(u,v\right)=0.$$

Thus, Equation (14) is reduced to

(20) $$\left\{\begin{array}{c} \partial_{t}u=F_{1}\left(u,v\right),\\ \partial_{t}v=F_{2}\left(u,v\right),\\ \partial_{x}u\left(-2.5\pi ,t\right)=\partial_{x}u\left(2.5\pi ,t\right)=\partial_{x}v\left(-2.5\pi ,t\right)=\partial_{x}v\left(2.5\pi ,t\right)=0, \end{array}\right.$$

with (x,t)∈[−5,5]×[0,0.75] and

(21) $$\left\{\begin{array}{ccc} F_{1}\left(u,v\right) & = & 0.1\partial_{x}^{2}u+3.6u^{1.5}-3.6u-0.229u^{1.5}\int_{-2.5\pi }^{2.5\pi }u\;dx+0.081\;\frac{u}{v^{2}+0.0215},\;\\ F_{2}\left(u,v\right) & = & 10\partial_{x}^{2}v+u-0.4v. \end{array}\right.$$

The training data, validation data and testing data are generated, based on (20), by applying a finite difference scheme to a 600 spatial mesh grid and then restricted to a 200 spatial mesh grid with the central difference scheme for $\Delta :=\partial_{x}^{2}$, and with a temporal discretization of the implicit Euler scheme, using a time step size of 0.01. Furthermore, we evaluate 14 different initial values, 10 of which were selected from a set of solutions with periodic patterns. The remaining initial values were generated by combining elementary functions. The reason for using different ways to produce initial values is to test if this method still works for periodical solutions.

We also add noise to the generated data in the following form:

$$u\left(x,y,t\right)=|u^{*}\left(x,y,t\right)+n_{l}Q_{1}\left|,\;v\left(x,y,t\right)=\right|v^{*}\left(x,y,t\right)+n_{l}Q_{2}|$$

where $n_{l}$ is the level of Gaussian noise added and $Q_{1}$ and $Q_{2}$ are random variables that follow from the normal distribution: $Q_{i}∼N\left(0,\sigma_{i}^{2}\right)$ for i=1,2, where $\sigma_{1}$ (or $\sigma_{2}$ resp.) is the standard deviation of $u^{*}$ (or $v^{*}$ resp.). The reason of imposing the absolute value sign is to avoid negative values, which may cause trouble to evaluate power functions with non-integer power, such as $u^{1.5}$.

We choose 15 blocks for the time on the interval [0,0.75] and denote $N_{Time}=15$. For spatial variables, we set $N_{Space}=200$, where $N_{Space}$ represents the number of space grids. Therefore, the dataset is

$$\left\{\left(u_{t,k},v_{t,k}\right):1\leq t\leq N_{Time},\;1\leq k\leq N_{Init}\right\},$$

where both $u_{t,k}$ and $v_{t,k}$ are matrices in $R^{N_{Space}\times N_{Space}}$. The following Table 5 and Table 6 show a summary of parameters for Frac-PDE-Net.

In [6], some assumptions are made on the model based on existing experimental knowledge of the biological behavior. For example, it is assumed that the operator $F_{2}\left(u,v\right)$ is linear in both *u* and *v*, while $F_{1}\left(u,v\right)$ is nonlinear in both *u* and *v*. As the form in (15),

$$F_{1}\left(u,v\right)=d_{1}\Delta u+P_{1}\left(u,v\right)+R_{1}\left(u,v\right).$$

In [6], the nonlinear dependence of $P_{1}\left(u,v\right)$ on *u* is via the combination of the power function $u^{\alpha }$ and the integration operator ∫udx, where α is further restricted to the range [1,2]. On the other hand, $R_{1}\left(u,v\right)$ is assumed to be linear in *u*, but nonlinear in *v* and the nonlinear dependence on *v* is via a fractional function whose denominator is a quadratic polynomial. Thanks to these a priori constraints, we consider the library $\left\{u,u_{x},u_{xx},v,v_{x},v_{xx},I,u^{\alpha }\right\}$ for ${\hat{F}}_{1}\left(u,v\right)$ and the library $\left\{u,u_{x},u_{xx},v,v_{x},v_{xx}\right\}$ for ${\hat{F}}_{2}\left(u,v\right)$, where α takes the form α=1.5+0.5sin(η) for η∈R to ensure that α∈[1,2].

The filters *q* are of size 1×19. The total number of parameters for approximating $F_{1}$ and $F_{2}$ is 29, and the number of trainable parameters in the moment matrices *M* is 32. To optimize the parameters, we again use the BFGS algorithm.

For this case, we added 1% noise to the generated data to form the observational data. The results are displayed in Table 7, in which the notations are consistent with those in Table 3.

Similar to the post hoc selection procedure we performed in Example 1, we also need to compare Model 1 (${\hat{F}}_{1}^{rsmPDE-Net}$) and Model 2 (${\hat{F}}_{1}^{PHrsmPDE-Net}$), and determine whether they differ significantly. Consider the time range to be [0,10] with time step size dt=0.05. Hence, there are 200 time grids which are denoted to be ${\left\{t_{i}\right\}}_{i=1}^{200}$, where $t_{i}=0.05i$, 1≤i≤200. At each time $t_{i}$, we introduce the residuals $E_{t_{i}}:=Y_{t_{i}}-Y_{t_{i}}^{*}$ and ${\tilde{E}}_{t_{i}}:={\tilde{Y}}_{t_{i}}-Y_{t_{i}}^{*}$, where $Y_{t_{i}}$ and ${\tilde{Y}}_{t_{i}}$ are associated to Model 1 and Model 2, respectively. We will test if the residuals ${\left\{E_{t_{i}}\right\}}_{i=1}^{200}$ and ${\left\{{\tilde{E}}_{t_{i}}\right\}}_{i=1}^{200}$ have similar distributions or not. Analogous to the previous case, we see from Table 7 that the coefficient in front of the term $\partial_{x}^{2}u$ in Model 2 (${\hat{F}}_{1}^{PHrsmPDE-Net}$) is a negative number -0.026, which leads to rapid concentration rather than diffusion effect. With this being said, Model 2 is essentially different from Model 1 and the distributions of ${\left\{E_{t_{i}}\right\}}_{i=1}^{200}$ and ${\left\{{\tilde{E}}_{t_{i}}\right\}}_{i=1}^{200}$ are totally different.

**Figure 6:**
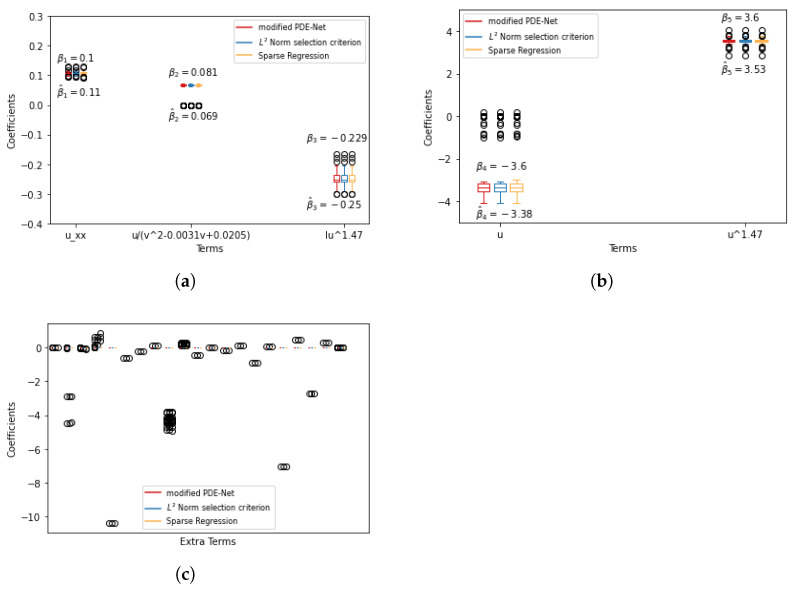
Simulation results for ${\hat{F}}_{1}\left(u,v\right)$ with 1% noise. (**a**) True positive discovering. (**b**) True positive discovering. (**c**) False positive discovering.

**Figure 7:**
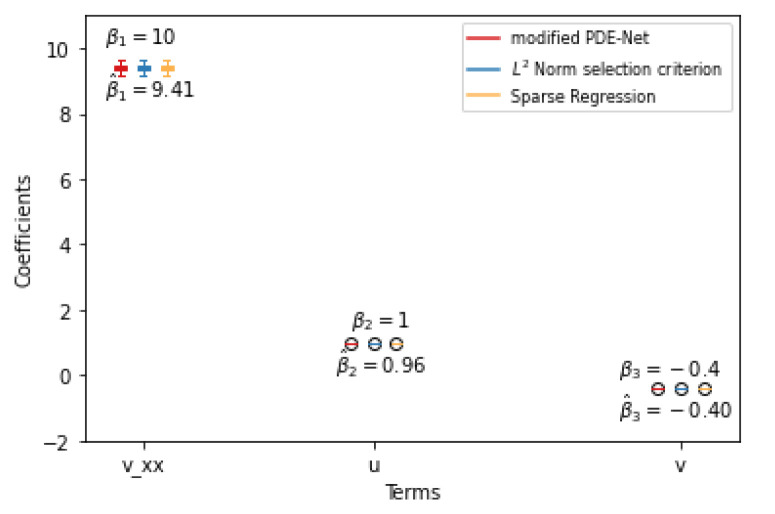
Simulation results for ${\hat{F}}_{2}\left(u,v\right)$ with 1% noise. True positive discovering.

To assess the stability of the results shown above, we repeated the experiments 100 times and the results are presented in Figure 6 and Figure 7. The plots show that there are some instances where the three methods fail to eliminate certain redundant terms. However, these instances are rare, as the median of these terms is 0, indicating that they appear infrequently.

## 4. Prediction

### 4.1. Example 1: The 2-Dimensional Model

In this section, we validate the robustness of the model discovered by Frac-PDE-Net in Example 1 by performing predictions with non-typical initial values $u_{0}$ and $v_{0}$,

$$u_{0}=\frac{50y^{2}-y^{4}+4}{800[1.2-\cos\left(\frac{\pi }{5}y\right)]}+4,\;v_{0}=\frac{1}{800}\left(50y^{2}-y^{4}+4\right)\left[2+\cos\left(\frac{\pi }{5}x\right)\right]+1.$$

We use the finite difference method to generate the “true data” in the forward direction using the known coefficients and terms in (16) and (17). The spatial step sizes (dx and dy) are set to $\frac{10}{64}$ and the time step size (dt) is $\frac{1}{1600}$. We then simulate the data using the trained model from Table 3 up to *t* = 0.5.

**Figure 8:**
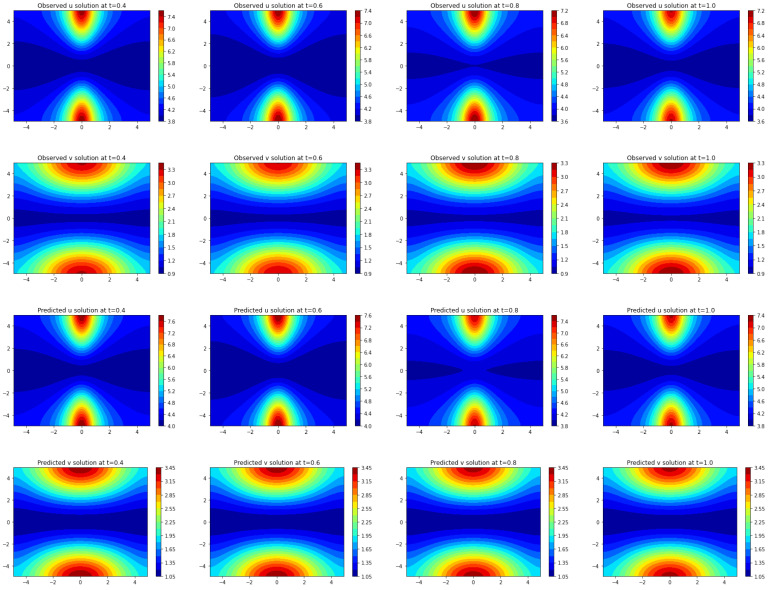
The first (second resp.) row shows the true dynamics of *u* (*v* resp.) at times t=0.4,0.6,0.8,and 1.0. The third (fourth resp.) row shows the predicted dynamics of *u* (*v* resp.) with 5% noise level using Frac-PDE-Net.

In Figure 8, both the true solution (u,v) and the predicted solution $\left(\tilde{u},\tilde{v}\right)$ of the trained model by Frac-PDE-Net are plotted at different time instances: t∈{0.4,0.6,0.8,1}. One can see from Figure 8 that the predicted solution is very close to the true one.

**Figure 9:**
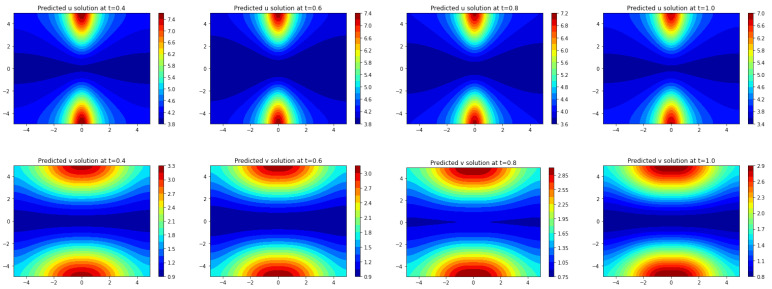
Images of the predicted dynamics using PDE-Net 2.0 with 5% noise level.

The results of the comparison between Frac-PDE-Net and PDE-Net 2.0 are presented in both graphical and quantitative form. The model discovered by PDE-Net 2.0 is shown in Table 8, while the predicted solutions are displayed in Figure 9. Although PDE-Net 2.0 only utilizes polynomials, the predicted images still have a similar shape to the true ones. To further evaluate the performance, the predicted errors are analyzed quantitatively using the $L^{\infty }$ norm and $L^{2}$ norm on the space domain [−5,5]×[−5,5], as seen in Table 9. The results show that Frac-PDE-Net has smaller errors compared to PDE-Net 2.0, highlighting its advantage.

### 4.2. Example 2: The One-Dimensional Model

In this section, we validate the robustness of the model discovered by Frac-PDE-Net in Example 2 in Section 3.2 by performing predictions with the following periodic initial values $u_{0}$ and $v_{0}$,

$$u_{0}\left(x\right)=0.0259+0.01\sin\left(3x\right),\;v_{0}\left(x\right)=0.06475+0.01\sin\left(3x\right).$$

**Figure 10:**
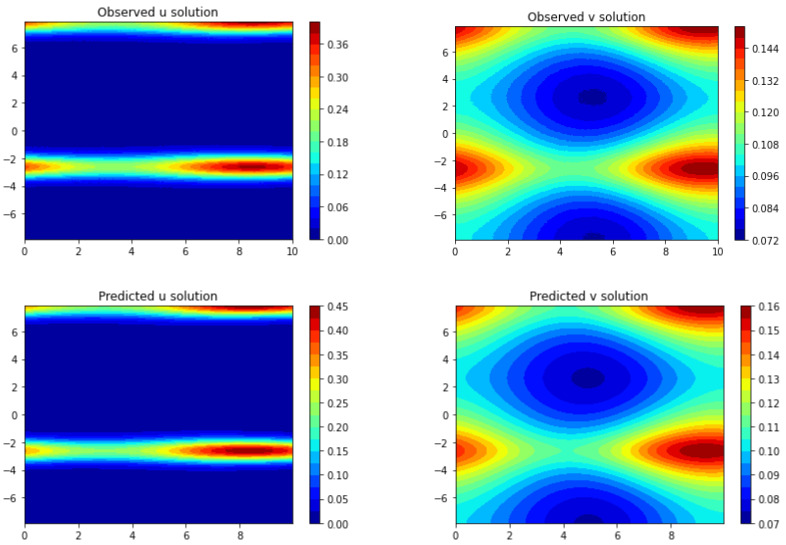
The first row shows the true dynamics of (u,v) for $\left(x,t\right)\in \left[-\frac{5\pi }{2},\frac{5\pi }{2}\right]\times \left[0,10\right]$. The second row presents the predicted dynamics of (u,v) with 1% noise level by Frac-PDE-Net.

We use finite difference method to generate the “true” data in the forward direction using the known coefficients and terms in (20) and (21). The spatial step sizes (dx and dy) are set to $\frac{5\pi }{200}$ and the time step size (dt) is $\frac{5}{100}$. The time interval considered is t∈[0,10]. We then simulate the data using the trained model from Table 7 over the time period [0,10]. In Figure 10, both the true solution and the predicted solution of the trained model by Frac-PDE-Net are plotted for t∈[0,10]. One can see from Figure 10 that the predicted solution is very close to the true one.

**Figure 11:**
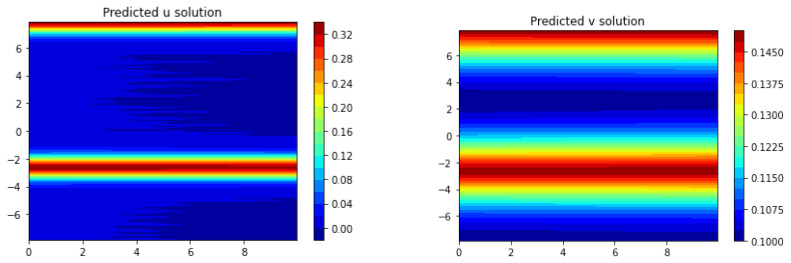
Images of the predicted dynamics of (u,v) for $\left(x,t\right)\in \left[-\frac{5\pi }{2},\frac{5\pi }{2}\right]\times \left[0,10\right]$ using PDE-Net 2.0 with 1% noise level.

The results of the comparison between Frac-PDE-Net and PDE-Net 2.0 are presented in both graphical and quantitative form. The model discovered by PDE-Net 2.0 is shown in Table 10, while the predicted solutions are displayed in Figure 11. We can clearly see that the predicted images by PDE-Net 2.0 are far from satisfactory compared to the true one in Figure 10. To further evaluate the performance, the predicted errors are analyzed quantitatively using the $L^{\infty }$ norm and $L^{2}$ norm on the space-time region $\left[-\frac{5\pi }{2},\frac{5\pi }{2}\right]\times \left[0,10\right]$ in Table 11. The results show that Frac-PDE-Net has much smaller errors compared to PDE-Net 2.0, highlighting its advantage.

## 5. Conclusions

Our approach, Frac-PDE-Net, builds on the symbolic approach developed in PDE-Net for addressing the discovery of realistic and interpretable PDE from data. While the neural network remains very efficient for generating and learning dictionaries of functions, typically polynomials, we have shown that if we enrich the dictionaries with large families of functions (typically uncountable), an extra-care is needed for selecting the important terms by penalization and by evaluating and testing the impact of a reaction term in the predicted solution. Quite remarkably, we can extract a sparse equation with readable terms and with good estimates of the associated parameters.

The introduction of rich families of functions, such as fractions (rational functions) is often necessary because they are well used by modelers, but also they can avoid the limitations of the approximation capacity of polynomials. Indeed, it might be necessary to have numerous terms in the expansion in order to have a correct approximation of the unknown reaction terms. As a matter of fact, we have introduced a very flexible family of fractions that avoid truncation based on powers $u^{p},v^{q},q,p\in N$. While we learn then the numerator and denominator coefficients in R, our approach is incorporated seamlessly in the symbolic differentiable neural network framework of PDE-Net by the introduction of extra layers.

Our work is originally motivated by the discovery and estimation of reaction–diffusion PDEs, with possibly complex terms such as fractions, non-integer powers, or non-local terms (such as an integral), as it has been introduced for the pollen tube growth problem [6]. Nevertheless, our selection approach could be used to handle other dictionaries, or in the presence of advection terms as our methodology does exploit the reaction–diffusion structure only for imposing some constraints on the dictionaries of interest, and because of the interpretability of each term in that case. As the next steps, the Frac-PDE-Net methodology can be improved by considering more advanced numerical schemes in time discretization, say implicit Euler or second-order Runge–Kutta. In that case, we expect to have a better accuracy and stability for model recovery and prediction. Another possible improvement would be to enrich the dictionaries of fractionals by replacing the current form N(u,v)=g(u)h(v) by more rational functions with denominators that depends both on *u* and *v*, say $N\left(u,v\right)=\frac{u-v}{u^{2}-v^{2}+1}$. Finally, we put an emphasis on the fact that Frac-PDE-Net reaches a trade-off by discovering the main terms of the PDE, accurately estimating each coefficient in order to gain interpretability, while it also allows effective long-term prediction, even for unseen initial conditions.

## Figures and Tables

**Table 1:** Fixed parameters for Frac-PDE-Net.

Parameter	Value
t	[0, 0.15]
dt	0.01
x & y	[−5, 5]
dx & dy	$\frac{10}{64}$
$N_{\mathrm{Init}}$	12
$N_{Time}$	15
$N_{Space}$	64

**Table 2:** Hyper-parameters selected by validation procedure Section 2.3 for Frac-PDE-Net.

Parameter	Value
$\lambda_{M}$ (5% noise level)	$3.28\times 10^{-5}$
$\lambda_{S}$ (5% noise level)	$4.93\times 10^{-5}$

**Table 3:** PDE model discovery with 5% noise.

True $F_{1}^{*}$	$0.3\Delta u+1-u-0.5\frac{uv}{u^{2}+0.25u+0.25}$
${\hat{F}}_{1}^{mPDE-Net}$	$0.305\Delta u+0.992-0.988u-0.510\frac{uv}{u^{2}+0.259u+0.265}$
	$-0.003\frac{v}{u^{2}+0.259u+0.265}+0.003v$
${\hat{F}}_{1}^{smPDE-Net}$	$0.305\Delta u+1.00-0.981u-0.532\frac{uv}{u^{2}+0.259u+0.265}$
${\hat{F}}_{1}^{rsmPDE-Net}$ (Frac-PDE-Net)	$0.304\Delta u+0.975-0.982u-0.501\frac{uv}{u^{2}+0.256u+0.260}$
${\hat{F}}_{1}^{PHrsmPDE-Net}$	$0.278\Delta u+0.969-0.993u-0.514\frac{uv}{u^{2}+0.301u+0.271}$
True $F_{2}^{*}$	$0.4\Delta v+0.4-0.2v-0.5\frac{uv}{u^{2}+0.25u+0.25}$
${\hat{F}}_{2}^{mPDE-Net}$	$0.398\Delta v+0.412-0.195v-0.510\frac{uv}{u^{2}+0.254u+0.263}$
	$-0.005\frac{v}{u^{2}+0.254u+0.263}-0.010u$
${\hat{F}}_{2}^{smPDE-Net}$	$0.398\Delta v+0.424-0.199v-0.542\frac{uv}{u^{2}+0.254u+0.263}$
${\hat{F}}_{2}^{rsmPDE-Net}$ (Frac-PDE-Net)	$0.400\Delta v+0.385-0.202v-0.490\frac{uv}{u^{2}+0.243u+0.256}$
${\hat{F}}_{2}^{PHrsmPDE-Net}$	0.344Δv+2.116−0.815v

**Table 4:** Hypothesis tests with 5% observation noise.

$H_{0}^{\left(i\right)}$ vs. $H_{A}^{\left(i\right)}$, 1≤i≤50	Number of Rejections
Bonferroni	49
Holm	49
B-H	49

**Table 5:** Fixed parameters for Frac-PDE-Net.

Parameter	Value
t	[0, 0.75]
dt	0.05
x	[−2.5π, 2.5π]
dx	$\frac{5\pi }{200}$
$N_{\mathrm{Init}}$	14
$N_{Time}$	15
$N_{Space}$	200

**Table 6:** Hyper-parameters selected for Frac-PDE-Net by the validation procedure as in Section 2.3.

Parameter	Value
$\lambda_{M}$ (1% noise level)	$1.88\times 10^{-7}$
$\lambda_{S}$ (1% noise level)	$1.62\times 10^{-6}$

**Table 7:** PDE model discovery with 1% noise level.

True $F_{1}^{*}$	$0.1\partial_{x}^{2}u+3.6u^{1.5}-3.6u-0.229u^{1.5}\int_{-2.5\pi }^{2.5\pi }u\;dx$
	$+0.081\;\frac{u}{v^{2}+0.0215}$
${\hat{F}}_{1}^{mPDE-Net}$	$0.118\partial_{x}^{2}u+3.959u^{1.361}-3.871u$
	$-0.223u^{1.361}\int_{-2.5\pi }^{2.5\pi }u\;dx+0.0749\;\frac{u}{(v+0.005{)}^{2}+0.0211}$
	$0.0002\;\frac{uv}{(v+0.005{)}^{2}+0.0211}-0.0029v$
${\hat{F}}_{1}^{smPDE-Net}$	$0.117\partial_{x}^{2}u+3.893u^{1.361}-3.976u$
	$-0.223u^{1.361}\int_{-2.5\pi }^{2.5\pi }u\;dx+0.0750\;\frac{u}{(v+0.005{)}^{2}+0.0211}$
${\hat{F}}_{1}^{rsmPDE-Net}$ (Frac-PDE-Net)	$0.0899\partial_{x}^{2}u+3.441u^{1.508}-3.363u-0.244u^{1.508}\int_{-2.5\pi }^{2.5\pi }u\;dx$
	$+0.0714\;\frac{u}{(v+0.0002{)}^{2}+0.0209}$
${\hat{F}}_{1}^{PHrsmPDE-Net}$	$-0.026\partial_{x}^{2}u+0.628u^{1.500}-2.333u+0.0393\frac{u}{(v-0.0479{)}^{2}+0.0154}$
True $F_{2}^{*}$	$10.0\partial_{x}^{2}v+u-0.4v$
${\hat{F}}_{2}^{mPDE-Net}$	$9.388\partial_{x}^{2}v+0.963u-0.400v$
${\hat{F}}_{2}^{smPDE-Net}$	$9.388\partial_{x}^{2}v+0.963u-0.400v$
${\hat{F}}_{1}^{rsmPDE-Net}$	$9.588\partial_{x}^{2}v+0.969u-0.403v$
${\hat{F}}_{1}^{PHrsmPDE-Net}$	$8.145\partial_{x}^{2}v+0.937u-0.387v$

**Table 8:** PDE model discovered by PDE-Net 2.0.

	Predicted Terms by PDE-Net 2.0 with 5% Noise
${\hat{F}}_{1}\left(u,v\right)$	0.0457Δu−1.765u+0.0938v+0.0008
${\hat{F}}_{2}\left(u,v\right)$	$0.243\Delta v-0.604u-0.277v+7(10^{-5})$

**Table 9:** Errors of predicted solutions for u and v by Frac-PDE-Net and PDE-Net 2.0.

	Noise	Frac-PDE-Net				PDE-Net 2.0			
$|\tilde{u}-u|$		t=0.4	t=0.6	t=0.8	t=1	t=0.4	t=0.6	t=0.8	t=1
$L^{\infty }$	5%	0.007254	0.010806	0.014310	0.017765	0.227365	0.331438	0.429602	0.522157
$L^{2}$	5%	0.000106	0.000158	0.000209	0.000260	0.002720	0.003986	0.005192	0.006341
$|\tilde{v}-v|$		t=0.4	t=0.6	t=0.8	t=1	t=0.4	t=0.6	t=0.8	t=1
$L^{\infty }$	5%	0.001503	0.002247	0.002988	0.003725	0.200241	0.293939	0.383577	0.469314
$L^{2}$	5%	0.000022	0.000033	0.000044	0.000054	0.001989	0.002930	0.003836	0.004708

**Table 10:** PDE model discovered by PDE-Net 2.0.

	Predicted Terms by PDE-Net 2.0 with 1% Noise
${\hat{F}}_{1}\left(u,v\right)$	$0.0001\partial_{x}^{2}u-3.95(10^{-5})u-6.05(10^{-5})v-0.0002$
${\hat{F}}_{2}\left(u,v\right)$	$5.22(10^{-5})\partial_{x}^{2}v+1.70(10^{-5})u+8.19(10^{-6})v+4.59(10^{-5})$

**Table 11:** Errors of predicted solutions for *u* and *v* by Frac-PDE-Net and PDE-Net 2.0.

	Noise	Frac-PDE-Net	PDE-Net 2.0
$|\tilde{u}-u|$		t∈[0,10]	t∈[0,10]
$L^{\infty }$	1%	0.062771	0.117773
$L^{2}$	1%	0.000029	0.000060
$|\tilde{v}-v|$		t∈[0,10]	t∈[0,10]
$L^{\infty }$	1%	0.009434	0.039400
$L^{2}$	1%	0.000010	0.000056

## Data Availability

Not applicable.

## Appendix A

### Appendix A.1. Term Combination after Simulation

During the process of simulation, if only the addition and the multiplication operators are involved, then it is not an issue to combine terms as the program can easily identify same terms and then add their coefficients together. However, combining similar terms can be difficult when fractional terms are present. To address this issue, we classify the simulation results into various groups before combining them.

As an example, we consider the scenario where the nonlinear term takes the form g(u)h(v), and one of the following two structures is assumed.

(i) *g* is linear and *h* is a fractional function whose denominator is a second order polynomial:
  $$\left(u+c_{1}\right)\;\frac{\alpha_{1}v+\alpha_{2}}{v^{2}+\beta_{1}v+\beta_{2}}.$$
(ii) *h* is linear and *g* is a fractional function whose denominator is a second order polynomial:
  $$\frac{\alpha_{3}u+\alpha_{4}}{u^{2}+\beta_{3}u+\beta_{4}}\;\left(v+c_{2}\right).$$

Therefore, the outcomes have 32 possibilities if we only classify terms and signs:

- Numerator (4 possibilities): *u*, *v*, uv, 1.
- Denominator (2 possibilities): quadratic function in *u* or *v*.
- Signs (4 possibilities): the sign of $\beta_{1}$ or $\beta_{2}$ can be either positive or negative.

There are now 32 groups. In each of them, all members share the same main terms and same signs in the denominator while the coefficients are allowed to be different. For example, in the group with the form

$$\frac{\alpha_{1}uv}{v^{2}+\beta_{1}v+\beta_{2}},$$

all members share the same term uv in the numerator, same terms $v^{2}$ and *v* in the denominator, and same signs of $\beta_{1}$ and $\beta_{2}$, while the specific values of $\alpha_{1}$, $\beta_{1}$ and $\beta_{2}$ may vary.

Based on the above groups, we will adopt the following general principle to proceed. If two terms live in distinct groups, then they are considered to be different and will not be combined. If two terms live in the same group, then we will further quantify how close their coefficients in the denominator (say $\beta_{1}$ and $\beta_{2}$) are. If these coefficients are close enough, then we will regard them as the “same” term and combine them by adding their coefficients in the numerator (say $\alpha_{1}$) together. So, the next question is how to quantify the distance between two members in the same group with possibly different coefficients (say $\beta_{1}$ and $\beta_{2}$).

We will illustrate the criterion in the following by studying a specific form $\frac{uv}{v^{2}+\beta_{1}v+\beta_{2}}$. More precisely, suppose there are two terms $T_{1}$ and $T_{2}$ as below,

$$T_{1}=\;\frac{\alpha_{1}^{\left(1\right)}uv}{v^{2}+\beta_{1}^{\left(1\right)}v+\beta_{2}^{\left(1\right)}},\;T_{2}=\frac{\alpha_{1}^{\left(2\right)}uv}{v^{2}+\beta_{1}^{\left(2\right)}v+\beta_{2}^{\left(2\right)}},$$

then we define their distance to be

(A1) $$D\left[T_{1},T_{2}\right]=\max_{i=1,2}\left\{\frac{|\beta_{i}^{\left(2\right)}-\beta_{i}^{\left(1\right)}|}{\max\{|\beta_{i}^{\left(2\right)}|,|\beta_{i}^{\left(1\right)}|\}}\right\}.$$

According to this concept, we combine $T_{1}$ and $T_{2}$ together if and only if $D[T_{1},T_{2}]<0.2$, that is when the relative difference between the coefficients is less than 0.2. In such a case, we add the coefficients $\alpha_{1}^{\left(1\right)}$ and $\alpha_{1}^{\left(2\right)}$ to obtain

$$T_{1}+T_{2}\approx T^{*}:=\alpha^{*}\frac{uv}{v^{2}+\beta_{1}^{\left(*\right)}v+\beta_{2}^{\left(*\right)}},$$

where

$$\alpha^{*}=\alpha_{1}^{\left(1\right)}+\alpha_{1}^{\left(2\right)},\;\beta_{1}^{\left(*\right)}=\frac{1}{2}\left[\beta_{1}^{\left(1\right)}+\beta_{1}^{\left(2\right)}\right],\;\beta_{2}^{\left(*\right)}=\frac{1}{2}\left[\beta_{2}^{\left(1\right)}+\beta_{2}^{\left(2\right)}\right].$$
